# The Institutional Worker

**Published:** 1912-11-30

**Authors:** 


### INSTITUTIONAL WORKER

Being a Special Supplement to "The Hospital"

### BUREAU OF INFORMATION.

### Rules for Correspondents.

1. Every letter, on whatever subject, must be accompanied by the coupon to be cut from the back cover (inside page) of The Hospital, current issue, and must contain the name and address of the correspondent with pseudonym for publication if desired.

2. Replies by post cannot be given, save under exceptional circumstances at the Editor's discretion.

3. Letters from Approved Homes in reply to special needs published in the Bureau should state terms and full particulars, and be sent prepaid under cover to the Editor of the Bureau with name written across coupon for identification.

4. Proprietors of Homes which have not yet been entered on the List of Approved Homes, but have spare accommodation likely to suit special needs, are invited to write for an application form for registration. The fee for registration, which includes two announcements of the Home in the Bureau, is 5s.

5. Special needs of every description relating to those who are sick or in difficulties are made known in these columns free of charge.

6. All communications to be addressed to The Editor of The Hospital, 28 Southampton Street, Strand, London, W.C., and marked "Bureau of Information."

### Approved Homes.

Note.—*No Home is added to the List without direct medical and other testimony to its good management.*

**Harrow-on-the-Hill.**—78 Butler Road. Principals: Mr. and Mrs. Stanley Gilbert. Electrical treatment, massage, and baths, for one patient only, under trained medical electrician and masseur, with latest apparatus. Domestic comfort studied. One or two non-resident patients can be treated.

**Leatherhead.**—1 Sackville Villas. Principal: Mrs. Day. Slight mental or convalescent cases. Good house, with garden. Cheerful companionship. Moderate terms.

**Royal Leamington Spa.**—Warneford House, Radford Road, and 37 Clarendon Square. Principal: Mrs. Ward, M.I.H. Medical, surgical, maternity, rest-cure, and convalescent. Special advantages for elderly people and permanent cases.

### Homes Wanted.

**For Child with Asthma.**—The patient is a little girl of eight, who has suffered from asthma from birth and is not improving in health. A Home in healthy country surroundings is needed, where she could have care at moderate terms. Replies to F. P. (136a).

**For Elderly Lady.**—The patient who needs accommodation in a Home is about seventy-two. She is quite well physically, but would require a certain amount of looking after. She has been living alone, and does not take proper care of herself. Her means are very limited. Prepaid replies forward to Enquirer (138a).

### Needs and Helpers.

**Incurable Home for Child.**—Can admission be procured to a Home for a girl, aged eleven, who is suffering from spina rigida? Her mother is intemperate. The father can pay 10s. a week. Replies to Matron (137x).

**Nursing Treatment for Rheumatic Patient.**—Dressmaker, suffering from rheumatism after colitis and quite helpless, needs six weeks' light treatment with electric baths to effect cure, by medical recommendation. Only very low terms can be paid. Can any reader offer help for this case? Replies to Hope (139x).

### Training and Employment.

**Hospital Experience.**—We note that you are a trained institutional worker, an expert organiser of laundry work, and that you want to take a few months' training in nursing in a well equipped hospital. We shall be pleased to put you in communication with any matron willing to offer you this advantage in return for services. You will find in "How to Become a Nurse" (The Scientific Press) the names of many hospitals where paying pupils are received for short periods.—N. E.

### Nursing in the Empire and Abroad.

**Nursing in Peking.**—There is a demand for trained British nurses, but it is very irregular; still there would be fairly continuous employment for at least one nurse among the Europeans. The fees are £5 a week plus board and washing. If you want to go out, write to the London Mission Hospital, Peking.—S. T.

**A Post in Penang or Singapore.**—The demand for private nurses in both these localities is fully met by the Colonial Nursing Association, and as you have full training, you should apply to the Secretary, Imperial Institute, South Kensington, and state your wish to be employed in that region. The Singapore Nursing Association employs three nurses for private work, and the Penang Nursing Association one under the C.N.A. Quite impracticable to go out on your own account.—Manwelho.

### Letter-Box.

**The Editor begs to acknowledge the receipt of communications, with enclosures, from:**—Matron, Wimbledon; the Misses E., Hemel Hempstead; Mrs. W. Bowdon.

**Communications have been received and attended to from:**—Miss O. J., Bowes Park; J. H. B., Carlisle; Rev. J. W., Penygroes; L. J. L., Brockley; Mrs. M., Lee; Mrs. L., Stranraer, N.B.; Miss A. G. S., Gorleston-on-Sea; Nurse E. F., Minehead; Miss A. J. H., Croydon; Mrs. V. W., New Radnor; Miss L. S., Clapham; Mrs. T., Gorleston-on-Sea; Nurse M., Pontesbury; Mr. S. G., Harrow.

**The Editor is much indebted for testimonials received in response to inquiries from:**—Mrs. G., Belfast; Miss B., Thame; Dr. J. S., Welbeck Street; Miss S., Colchester; Dr. B., Clacton-on-Sea; Miss M., Hemel Hempstead.

E. N. (134a).—We note that you have made choice of the Home you require and that no more replies are needed.

Missioner.—Very pleased to comply with your request, and shall be happy to be of service in your work if occasion offers.

Government Medical Officer, Wei-Hai-Wei; Captain T. M. S., Baghdad; H.B.M. Consul, Grand Canary; Acting B. Consul-General, Valparaiso.—Hearty thanks for information supplied.

Nurse McC. and Miss L. S.—The address given was only a house-of-call, but it was quite genuine. See above.

Nurse F. M. M.—Will do our utmost to help you. Let us have full particulars of any alterations you may make.

Nurse D.—You must write yourself to any likely cases of which particulars are given in our columns. See Rule 3.

**Persons requiring nurses and nurses requiring appointments will find their needs met in the adjoining pages, where many useful announcements appear this week.**

### INSTITUTIONAL HOUSEKEEPING.

### Readers' Questions with Practical Answers.

**Carving Off the Table.**—Your housekeeper should superintend the carving at the side, even if she does not with a large party undertake the whole work herself. We prefer the joints to make an appearance in the room, because portions cut in the kitchen very easily grow cold, and are not in any case nearly so appetising as when quite freshly cut from the joint. For some unexplained reason, also, there is apt to be dissatisfaction with the quality of the meat when the joint is not visible to proclaim itself. The carving is a most important branch of the commissariat, and if you have no one on the staff who is an expert at it, you should at once take steps to remedy this state of things by getting someone trained to undertake it at a school of cookery.—A. F.

**Taking Turns at the Housekeeping.**—Your plan of allowing the nurses to take turns at the housekeeping is in practice disastrous to the comfort of the household and the ruin of any system of economy. It is very doubtful, moreover, whether it can impart a sound knowledge of household economics to those engaged in the costly series of experiments it involves. The result is that with the interested competent nurse the staff lives in clover, while under the uninstructed nurse, who dislikes housekeeping and knows nothing about the handling of food, everyone is half starved. Even a change of matron makes an astonishing difference sometimes in the character of the food procurable for a given allowance. In one hospital known to us, where a weekly allowance was paid per head, the food was not only plentiful and excellent under a capable matron, but enough was saved in a short period to buy a piano for the staff and lay out a tennis court. The management changed, and under the new head the allowance proved barely sufficient to provide mere necessaries, yet no one was the gainer. This was in the Colonies, and it may serve to illustrate the folly of expecting to keep up a high standard of catering under a constant succession of novices trying to govern when they ought to be humbly learning the A B C of housekeeping.—R. T. (Leicester).

**Bright Table Glass.**—It is by no means necessary to go to a great deal of expense to make the table look well in regard to its best ornament, clear and shining glass. The tumblers you buy should not be too thick, and it is better to have them unornamented, as they are more easily replaced when broken. It is essential to a good table that they should all match. Their washing is an important matter, and you cannot do better than give the maid responsible for this work the following directions: All glasses should be washed in warm soapy water, rinsed in cold water, dried on soft linen cloths, and polished at once on a second dry cloth. Those which have contained milk must first soak in cold water, and not be washed with the others. Any glasses that may have got into bad condition through neglect can be brightened by adding a few drops of ammonia to the water, and stains from bottles can be removed by half filling the bottle with torn-up newspaper, filling it with water, and then allowing it to stand for twenty-four hours. The printer's ink dissolves the stain. But if the process of removing the pulp afterwards be found too troublesome, try vinegar, with either salt or tea-leaves. Do not omit to harden all new tumblers by placing them in a pan of cold water and bringing it slowly to the boil, allowing them to remain in till the water cools.—Holy Poverty.

**Dishes Without Meat—Vegetable Pie.**—You will find this a savoury and nourishing dish. Butter a pie-dish and cover the bottom with sliced tomato. Slice two onions fine and fry them in an ounce of butter. Place a layer of hard-boiled egg over the tomato, sprinkle with pepper and salt, chopped parsley, a little of the onion, and cover with Bêchamel sauce; have ready 2 oz. of cooked vermicelli, put a layer over the sauce, continue with the layers of tomato, egg, onion, and vermicelli until the dish is full, cover with mashed potato, and put in the oven to brown.—Weary Willie.

**Use for Warm Blankets.**—Try making up your old blankets into warm quilts. Place two or three together and cover them with a pretty flowered sateen. Small eyelet holes should be cut through at intervals, as in a ventilated down quilt, and button-holed round with strong silk thread, either the colour of the sateen or a contrasting colour. This gives ventilation and also keeps the layers of blanket in place. If it is desired to make them more ornamental a frill of either plain sateen to match the body colour of the flowered or of the same material could be laid between the edges and stitched in. These make warm covers for convalescents who are allowed to sit or lie outside.—Parsimony.

**Candle-ends.**—Do not throw these away. An excellent and economical floor and furniture polish can be made by putting all the ends left in the candlesticks into turpentine. A paraffin tin cut in half makes an excellent receptacle. Put in the turpentine, say, a gallon, and a little paraffin with it, and throw into it the candle-ends every time they are taken out. The turpentine will dissolve the wax and the wicks will drop to the bottom. When it has the consistency of rather soft dripping it is fit to use. A very little is required, and the polish is brilliant.—Anne.

**To Polish Linoleum.**—You will find soft soap very good for your linoleum. The floor should be washed over with a wet flannel, on which the soft soap is rubbed, and polished with a dry flannel immediately. This not only takes the dirt off, but leaves a soft polish which is not so slippery as that produced by wax.—Still-room.

**Cheap Material for Swabs and Rubbers.**—Try buying sheep cloths, the casings in which frozen meat is sent over. "Sheep shirts" they are technically called. Butchers sell them for about 1½d. to 2d. each. They are beautifully soft and absorbent, and contain a quantity of material. I conclude you only need them for domestic purposes. Wash and boil them thoroughly, and tear up. They make excellent straining cloths for soups, etc.—Matron.

**A Use for Stale Bread.**—If you grind up the stale bread and put it away in bottles you will find it most useful for puddings where dry breadcrumbs are required. Stale bread—crusts and all—if soaked over night in cold milk and water makes the most delightfully light boiled puddings. The bread must be squeezed free of the liquid before using. Stuffing made of this instead of dry breadcrumbs remains moist, and is more delicate.—Colonial.

NOTICE TO OUR READERS.

The Editor welcomes for Institutional Housekeeping any contribution from those engaged in the work of the commissariat in institutions. All accepted contributions are paid for. Queries answered on any subject relating to the supply, cost, storage, distribution, or preparation of food.

### Editor's Notices.

**Contributions:**

Contributions should be written, or preferably typed, on one side of the paper only, and all articles sent in are accepted upon the distinct understanding that they are forwarded to The Hospital only.

The Editor cannot undertake to return MSS. not used, but when a stamped directed envelope is enclosed the MSS. may be returned if a special request is made.

Accepted articles and paragraphs of news will be paid for after publication at the scale rate.

**Address:**

To prevent delay all contributions and letters on editorial business must be addressed exclusively to the Editor, "The Hospital," 29 Southampton Street, Strand, London, W.C. It is important that this regulation shall be strictly observed.

**Correspondence:**

Correspondence on all subjects is invited. The name and address of correspondents must be given as a guarantee of good faith, but not necessarily for publication.

**Special Articles:**

Special articles are invited, and questions, inquiries, and paragraphs upon all matters relating to the work, administration, and management of general, special, mental, fever, cottage and convalescent hospitals, sanatoria, homes, institutions, societies and organisations for the treatment or care of the sick, injured and dependants of all classes. Every matter affecting the interests and welfare of the staff of all grades working in these institutions will receive special consideration.

**Administrative Medicine, Research and Items of News:**

Special terms will be paid for approved articles on subjects in this wide field by experts who have made some section of it their special study and interest. We wish to give prominence to every movement tending to advance accuracy of diagnosis, efficiency in treatment, and the development of modern methods to eradicate disease and promote the welfare of the suffering.

**A Bureau of Information:**

We invite inquiries and applications for information and help in providing for that numerous class of sufferers who require special care or house-room which they cannot obtain in their own homes or secure for themselves. **We cannot, however, prescribe, or recommend practitioners.**

**Books for Review:**

Publishers are particularly requested to send advance proofs of any new books of importance whenever possible, as the Editor has made arrangements to publish immediate reviews on a new plan.

**Photographs, Plans, Blocks, and Illustrations:**

It is requested that wherever possible MSS. may be accompanied by illustrations in any of the above forms. The name of the sender and of the article to which it belongs should be written on the back of each photograph, drawing, or block for purposes of identification.

**Local Papers:**

Newspapers containing reports or news paragraphs should be marked and addressed to the Sub-Editor.

**The Coupon System:**

A coupon will be found at the bottom of the third inside page of the cover each week. This coupon must be attached to every question or inquiry to which an answer is desired in The Hospital.

### Our Purpose.

It is our intention to render this section of The Hospital of the greatest possible service to the Institutional Worker. The term is employed in its widest sense, and includes every individual engaged in or associated with Charitable, Poor-Law, and Municipal Administration, as well as the Medical, Health, and Sanitary Services.

Although this Supplement was primarily intended to be of assistance to those seeking appointments, and numerous announcements of vacancies appear in its columns every week, it is felt there are many other ways in which it can be of even greater value. Our readers will observe that this week a number of pages have been added containing information which cannot fail to be of assistance to them in the work.

Our purpose is to make "The Institutional Worker" of practical utility to everyone connected with or attracted to Institutional life, and in this you may help us and yourself by showing a lively interest in this section and offering such suggestions for its improvement as may occur to you from time to time.

### Manager's Notices.

**All letters on business matters must be addressed to The Manager, "The Hospital," 28 and 29 Southampton Street, London, W.C.**

**Subscriptions**, which may commence at any time, are payable in advance. Cheques and Post-Office Orders should be crossed London County and Westminster Bank, Covent Garden Branch, and made payable to the Manager of The Hospital.

Rates of Subscription (payable in advance).

| | | |
|---|---|---|
| UNITED KINGDOM. | | |
| Three Months (including Postage) | ... ... ... ... | 2s. 0d. |
| Six Months do. | ... ... ... ... | 4s. 0d. |
| Twelve Months do. | ... ... ... ... | 6s. 6d. |
| FOREIGN AND COLONIAL. | | |
| Three Months (including Postage) | ... ... ... ... | 2s. 6d. |
| Six Months do. | ... ... ... ... | 5s. 0d. |
| Twelve Months do. | ... ... ... ... | 8s. 8d. |

**Advertisements:**

To ensure insertion, all advertisements for The Hospital must reach the Manager not later than Wednesday at noon in each week. For scale of charges see pages v and 4.

**Special Rates** will be quoted for those institutions that agree to send all their public notices to The Hospital each year, so as to make it a file of such announcements for ready reference.

This form may be used for small prepaid advertisements, and when filled up should be posted to

*The Manager*, The Hospital,
28 and 29 Southampton Street,
Strand, W.C.

Appointments Wanted, 21 words ... 1s. 0d.
Appointments Vacant, 21 words ... 2s. 6d.

THE SCIENTIFIC PRESS, LTD., 28 & 29 Southampton Street, Strand, London, W.C.

**BOOKS FOR INSTITUTIONAL WORKERS.**

| | |
|---|---|
| Burdett's Hospitals and Charities . . . | 10s. 6d. |
| Hospital Expenditure: The Commissariat . . . | 2s. 6d. |
| Modern Nursing in Hospital and Home . . . | 2s. 6d. |
| Surgical Instruments and Appliances used in Operations . . . . . . . . | 1s. 8d. |
| Midwife's Pocket Dictionary . . . . | 1s. 1d. |
| Surgical Ward Work and Nursing . . . . | 5s. 5d. |

Ledgers, Account Books, and every kind of Institutional literature.

Of all Booksellers, or of The Scientific Press, Ltd., 28 & 29 Southampton Street, Strand, London, W.C.

### HOSPITAL EXPENDITURE: THE COMMISSARIAT.

A USEFUL HANDBOOK FOR HOSPITAL MANAGERS AND MATRONS.

Crown 8vo, bound in cloth boards, **2s. 6d.** post free. Of all Booksellers, or of the Publishers—

THE SCIENTIFIC PRESS, LIMITED, 28/29 Southampton St., Strand, London, W.C.

